# Preparation of HMX/PMMA Composite Microspheres with Excellent Properties by Photoinitiated Emulsion Polymerization

**DOI:** 10.3390/molecules31111911

**Published:** 2026-06-02

**Authors:** Shimin Zhang, Nan Wu, Kaixuan Jia, Xinyue Huang, Xudong Wu, Conghua Hou, Honglu Li, Jingyu Wang

**Affiliations:** 1 School of Environment and Safety Engineering, North University of China, Taiyuan 030051, China; 2 Lanshantou Subdistrict Office, Lanshan District, Rizhao 276808, China

**Keywords:** photoinitiated emulsion polymerization, safety performance, modifier, HMX, PMMA

## Abstract

High-energy insensitive energetic materials are currently a research focus. Octogen (HMX) is one of the best-performing nitramine explosives, but its poor crystal morphology causes high mechanical sensitivity, limiting its application. This study proposed a method combining spheroidization, nanosizing, and coating desensitization. Nano-SiO_2_ and TiO_2_ were used to modify methyl methacrylate (MMA), and HMX/PMMA composite energetic microspheres were successfully prepared with the assistance of an ultraviolet (UV) lamp for catalytic polymerization. Molecular dynamics simulations determined the optimal particle ratios, and the effects of modifier content on morphology, crystal form, thermal stability, mechanical properties, and static mechanical properties were experimentally investigated. The prepared HMX/PMMA/modifier microspheres exhibited uniform size, dense structure, excellent performance, and ideal coating. Thermal decomposition kinetics showed that the activation energy of HMX/PMMA/SiO_2_ (0.75 wt% SiO_2_) increased by 79.86 kJ/mol and 27.55 kJ/mol compared with raw HMX and HMX/PMMA, respectively. Its impact sensitivity was 3.6 times that of raw HMX, and its friction sensitivity was twice that of raw HMX. Static mechanical analysis revealed that the compressive strength of HMX/PMMA/SiO_2_ (0.75 wt% SiO_2_) and HMX/PMMA/TiO_2_ (0.5 wt% TiO_2_) microspheres increased by 7.3 MPa and 6.1 MPa, respectively, over HMX/PMMA, indicating significant improvement. Overall, HMX/PMMA/SiO_2_ and HMX/PMMA/TiO_2_ microspheres prepared by photoinitiated emulsion polymerization exhibited excellent thermal stability and mechanical properties.

## 1. Introduction

**Figure 1:**
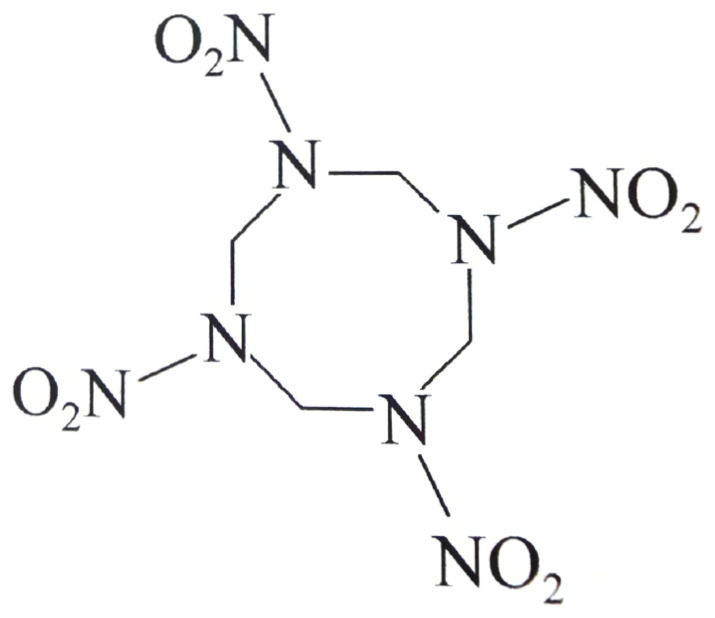
The chemical structure of HMX.

Octogen (HMX) is a common high-energy monomolecular nitramine explosive. Its chemical name is octahydro-1,3,5,7-tetranitro-1,3,5,7-tetrazocine; its molecular formula is C_4_H_8_N_8_O_8_; and the chemical structure of HMX is shown in Figure 1. Owing to its high energy density, good chemical stability, high thermal stability, and excellent detonation performance, it is widely used in military and civilian fields such as rocket propellants, missile warheads, and mine blasting [1]. However, HMX has poor mechanical sensitivity and is prone to accidental combustion or explosion when subjected to external energy stimulation, which may cause unnecessary economic losses or casualties during its production, storage, transportation, and use, thereby limiting its practical application [2,3,4,5]. Therefore, necessary measures must be taken to reduce the sensitivity of HMX and enhance its safety. Choosing a well-performing binder to form a protective layer on the exterior of energetic materials is a common and effective desensitization approach [5,6].

Poly (methyl methacrylate) (PMMA) is the substance/polymer formed by the spontaneous polymerization of methyl methacrylate (MMA). It has a linear polymer structure, smooth surfaces, good mechanical properties, and chemical stability [7,8]. This well-performing binder has been shown to be capable of coating energetic materials to enhance their safety [9]. Further research on PMMA has revealed that its surface is relatively easy to damage and sensitive to cracks and has low elasticity and high hardness. As a result, the composite microspheres after coating have poor mechanical properties and are difficult to compress and form. Therefore, it is necessary to modify PMMA to optimize the overall performance of the composite microsphere. Studies have shown that most researchers choose to add nanoparticles to PMMA to improve its mechanical properties, and different nanoparticles have different effects on enhancing the performance of composite materials. For example, Ralf Lach et al. [10] added nano-SiO_2_ to PMMA, and the prepared PMMA/SiO_2_ composite material exhibited enhanced crack resistance compared with pure PMMA. When the SiO_2_ content was 10 wt%, a significant improvement of approximately 70% was achieved compared with pure PMMA. Amit Chatterjee [11] added nano-TiO_2_ to the PMMA matrix; the incorporation of TiO_2_ nanofillers improved the thermal properties, mechanical properties, and UV absorption capacity of PMMA. The tensile modulus and dimensional stability of the composite material were increased, and the tensile modulus increased monotonically with the addition of TiO_2_. When the TiO_2_ content was 2%, the tensile modulus of the composite material increased by 35%.

To obtain HMX/PMMA composite energetic microspheres with excellent performance, nano-SiO_2_ and TiO_2_ were used as modifiers in this study. Nanoparticles possess characteristics such as small size, large specific surface area, high surface energy, excellent dispersibility, and strong interactions with the polymer matrix. Consequently, composites filled with nanoparticles exhibit superior physical and mechanical properties to pristine polymer materials [12]. Meanwhile, owing to the presence of nanoparticles, the mobility of polymer molecular chains is enhanced, thereby improving the binding energy between the hard and soft segments of the polymer and ultimately optimizing its mechanical properties, thermodynamic properties, and corrosion resistance [13,14,15]. In this way, the poor mechanical performance issue of HMX/PMMA composite microspheres is successfully addressed, which broadens the application scope of HMX-based composite materials.

This study combines simulation and experimental methods to investigate the effects of SiO_2_ and TiO_2_ on HMX/PMMA composite microspheres. Simulations predict the compatibility and mechanical properties of different systems and yield the optimal theoretical ratios. HMX/PMMA/SiO_2_ and HMX/PMMA/TiO_2_ composite microspheres were prepared by photoinitiated emulsion polymerization. They were analyzed and characterized using SEM, XRD, FT-IR, and DSC, and mechanical sensitivity tests were conducted using a BAM friction sensitivity tester and a BAM impact sensitivity tester. Finally, the samples were pressed into pellets to evaluate their static mechanical properties.

## 2. Molecular Dynamics

### 2.1. Model Construction

Molecular dynamics (MD) simulation is a computational technique based on Newton’s laws of mechanics, mainly used to simulate and analyze the microscopic behavior and properties of substances. By simulating the motion trajectories of atoms or molecules under force, this technique provides insights into the basic physical and chemical properties of materials [15]. In this section, molecular dynamics simulation was used to conduct an in-depth study on the atomic properties of the composite system with HMX as the substrate, PMMA as the binder, and modified SiO_2_ and TiO_2_ as modifiers.

Using the static module in Materials Studio, the main growth interface (0 1 1) of HMX was identified as the adsorption surface, and a supercell was constructed. Then, HMX, PMMA, SiO_2_, and TiO_2_ were placed in a periodic box according to a certain mass ratio to construct a supercell. The mass ratio of HMX to binder was M_HMX_:M_PMMA_ = 95:5, and the mass percentages of the modifier (SiO_2_ or TiO_2_) were 0.25%, 0.5%, 0.75%, and 1%, respectively. Then, the structure was geometrically optimized, and molecular dynamics simulations were performed using the COMPASS force field in the Forcite module. The binding energy in the HMX/PMMA/modifier system was calculated to determine the optimal proportion of each component in this composite energetic system.

### 2.2. Binding Energy

Binding energy is an analytical method used to describe intermolecular interactions. It is used not only to estimate the stability of materials but also to quantitatively characterize intermolecular forces. The greater the binding energy, the stronger the intermolecular forces within the formed composite system, the more stable the composite system, and the stronger the system’s compatibility [16]. The calculation formula for the binding energy *E_bind_* between the polymer binder, modifier, and HMX is as follows:

*E_bind_* = −*E_inter_* = −(*E_total_* − *E_explosive_* − *E_poly_*) (1)

where *E_bind_* is binding energy; *E_inter_* is the interaction energy; *E_total_* is the total energy of the equilibrium system; *E_explosive_* is the explosive energy of the energetic material after removing the binder; and *E_poly_* is the average single-point energy of the binder. The binding energy *E_bind_* is the negative value of the interaction energy *E_inter_*.

**Figure 2:**
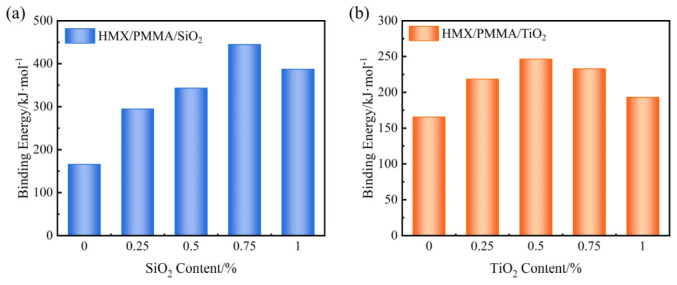
Binding energy of (**a**) HMX/PMMA/SiO_2_ and (**b**) HMX/PMMA/TiO_2_.

The pre-built HMX/PMMA/SiO_2_ and HMX/PMMA/TiO_2_ systems were structurally optimized under the COMPASS force field. Subsequently, molecular dynamics calculations were performed on the structures for 500 ps in the NVT ensemble (canonical ensemble, constant number of particles, constant volume, constant temperature) to allow full relaxation of the structures. During the simulation, the temperature was set to 298 K, and the Velocity Scale method was used for temperature control. The pressure was set to 10^−5^ GPa, and the Parrinello method was selected for pressure control. Finally, the equilibrium structures of each system were obtained for subsequent computational analysis. The calculation results are shown in Figure 2.

As can be seen from Figure 2, after adding the modifiers SiO_2_ and TiO_2_, the binding energy of the HMX/PMMA system increased. For the HMX/PMMA/SiO_2_ system, as the mass of SiO_2_ increased, the binding energy of the system first increased and then decreased, reaching its maximum when the SiO_2_ content was 0.75%. For the HMX/PMMA/TiO_2_ system, as the mass of TiO_2_ increased, the binding energy also first increased and then decreased, reaching its maximum when the TiO_2_ content was 0.5%, indicating the most stable system.

### 2.3. Mechanical Properties Analysis

To further analyze the effect of each modifier on the mechanical properties of the composite system after their addition, simulations were conducted on the mechanical parameters of SiO_2_ and TiO_2_ with different additive ratios, as shown in Table 1 and Table 2.

By comparing the mechanical property parameters of simulated systems with different composite ratios, critical insights into the composite system were elucidated. Following the incorporation of modifier SiO_2_, the tensile modulus, bulk modulus, and shear modulus of the system all exhibited an increasing tendency. The introduction of nano-SiO_2_ significantly enhanced the composite’s resistance to deformation under external pressure, thereby augmenting the material’s rigidity. Furthermore, the elevation in bulk modulus further fortified the material’s fracture resistance. The Cauchy pressure (C_12_-C_55_) was utilized to evaluate the ductility of the composite system, while the K/G ratio was employed to assess the material’s toughness. The K/G ratio is calculated as the quotient of the bulk modulus to the shear modulus. When the addition amount of modifier nano-SiO_2_ in the simulated system was 0.25%, 0.5%, and 0.75%, the system demonstrated enhanced toughness and superior ductility. In conclusion, at a SiO_2_ dosage of 0.75%, the mechanical property parameters of the simulated system were superior to those at other ratios, exhibiting improved mechanical performance. This observation is consistent with the findings regarding binding energy.

Identical mechanical property analysis conducted on the HMX/PMMA/TiO_2_ composite system revealed that the incorporation of modifier TiO_2_ improved the mechanical property parameters of HMX/PMMA to varying degrees. Since the differences between some parameters were minor, the Cauchy pressure and K/G ratio were adopted as the primary criteria for analysis. Combined with the analysis results of the binding energy between systems, it was concluded that the modification effect of the HMX/PMMA system was optimal when the TiO_2_ dosage was 0.5%.

## 3. Results and Discussion

### 3.1. Effect of Different Modifier Contents on Composite Microspheres

#### 3.1.1. Effect of Nano-SiO_2_ Content

**Figure 3:**
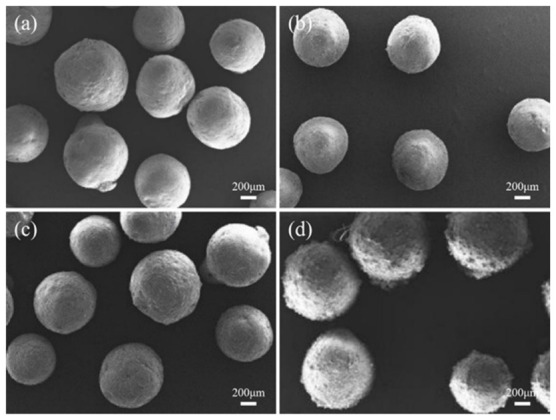
SEM images of HMX/PMMA/SiO_2_ composite microspheres under different SiO_2_ ratios of (**a**) 0.25%, (**b**) 0.5%, (**c**) 0.75%, (**d**) 1%.

Figure 3 shows the SEM of HMX/PMMA composite microspheres with different SiO_2_ ratios. As the SiO_2_ content increases, the surface roughness of the HMX/PMMA composite microspheres increases significantly, and the morphology gradually changes from relatively smooth to a rough surface with an obvious granular texture. Specifically, when the SiO_2_ content is 0.25% (Figure 3a), the microspheres exhibit a spherical morphology with slight surface undulations but are overall dense and smooth, indicating that a small amount of SiO_2_ is mainly distributed inside the PMMA matrix with limited surface coverage. When the content increases to 0.5% (Figure 3b), the microspheres still maintain good spherical morphology. When the content reaches 0.75% (Figure 3c), the microspheres have an intact morphology with a significant increase in surface roughness, indicating that the SiO_2_ particles are notably enriched on the surface of the microspheres. When the content increases to 1% (Figure 3d), the microsphere surface becomes extremely rough, and dense granular protrusions indicate that SiO_2_ covers the outer surface of the PMMA coating layer, exhibiting an obvious surface-modified structure. The morphological changes are attributed to the migration and enrichment of SiO_2_ nanoparticles from the interior of the polymer matrix to the surface as their content increases during the emulsion polymerization and coating process. Notably, SiO_2_ not only acts as a modifier for PMMA in the entire emulsion system but also serves as a stabilizer at the oil–water interface, regulating the surface tension between the emulsion and HMX, thereby enabling PMMA to spread more uniformly on the HMX surface. However, when the SiO_2_ content reaches 1%, it is difficult for the excess SiO_2_ to stably exist in water, which reduces emulsion stability and further exacerbates the surface roughness of the microspheres. Combined with the molecular dynamics simulation results, the composite system exhibits optimal binding energy and morphology when the SiO_2_ content is 0.75%. Therefore, 0.75% is determined as the optimal condition for preparing HMX/PMMA composite microspheres.

#### 3.1.2. Effect of Nano-TiO_2_ Content

**Figure 4:**
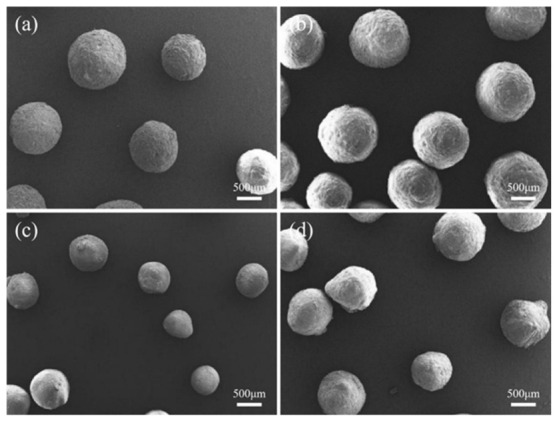
SEM images of HMX/PMMA/TiO_2_ composite microspheres under different TiO_2_ ratios of (**a**) 0.25%, (**b**) 0.5%, (**c**) 0.75%, (**d**) 1%.

The SEM of HMX/PMMA/TiO_2_ composite microspheres with different TiO_2_ ratios is shown in Figure 4. As the TiO_2_ content increases, the surface roughness and particle size distribution of the HMX/PMMA/TiO_2_ composite microspheres exhibit a non-monotonic change. Specifically, when the TiO_2_ content is 0.25% (Figure 4a), the microspheres have good sphericity, a relatively smooth and dense surface, good particle dispersion with no obvious agglomeration, and a relatively uniform particle size distribution. When the content increases to 0.5% (Figure 4b), the microspheres still maintain a regular spherical shape, with a certain degree of surface roughness arising from the attachment or embedding of a small amount of TiO_2_ particles on the PMMA matrix surface. When the TiO_2_ content increases to 0.75% (Figure 4c), the particle size of the microspheres decreases significantly and the distribution becomes uneven. When the TiO_2_ content reaches 1% (Figure 4d), the surface roughness of the microspheres increases markedly, presenting an obvious granular texture, indicating that TiO_2_ is enriched on the surface. Meanwhile, the sphericity decreases slightly, some particles show irregular deformation. Similar to SiO_2_, TiO_2_ not only acts as a modifier for PMMA but also serves as an oil–water interface stabilizer. At an addition amount of 0.5%, the HMX/PMMA/TiO_2_ emulsion system exhibited the best visual homogeneity among all formulations, yielding microspheres with regular morphology and dense structure. Combined with the molecular dynamics simulation results, the improvement in intermolecular binding energy and mechanical properties of the composite system is the most significant at this addition level. Therefore, 0.5% is determined as the optimal TiO_2_ content for preparing HMX/PMMA composite microspheres.

### 3.2. Morphology and Particle Size Analysis

**Figure 5:**
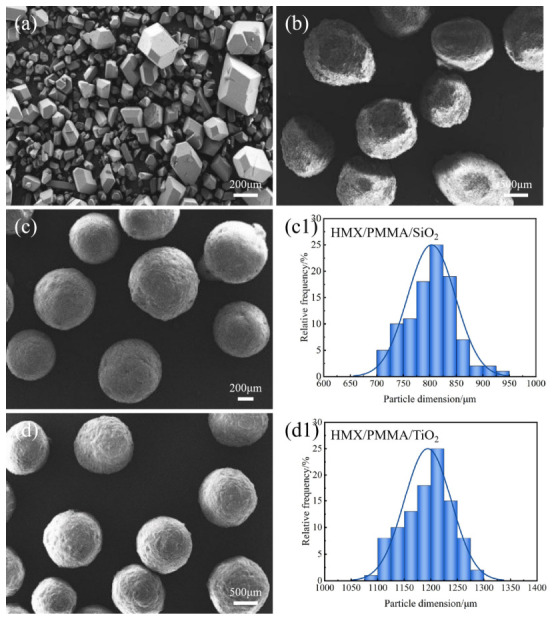
SEM images of (**a**) raw HMX, (**b**) HMA/PMMA, (**c**) HMX/PMMA/SiO_2_ (0.75%), (**d**) HMX/PMMA/TiO_2_ (0.5%), and particle size distribution of (**c1**) HMX/PMMA/SiO_2_ (0.75%), (**d1**) HMX/PMMA/TiO_2_ (0.5%).

The morphologies of raw HMX, HMX/PMMA, HMX/PMMA/SiO_2_, and HMX/PMMA/TiO_2_ composite microspheres were characterized using SEM. The result is shown in Figure 5. Raw HMX had a prismatic crystal shape with a wide particle size distribution. After HMX was coated with PMMA, spherical-like particles with rough surfaces were formed. In contrast, the composite microspheres modified with SiO_2_ and TiO_2_ exhibited dense and regular spherical structures, with good dispersibility between particles. Comparison with unmodified HMX/PMMA composite microspheres revealed that, after adding the modifiers SiO_2_ and TiO_2_, the modifier particles and PMMA jointly formed a coating layer, resulting in denser microspheres and more-uniform particle size. This is because, after modifying the nanoparticles with the coupling agent MPTMS, C=C groups were grafted onto the surface of the nanoparticles, improving their lipophilicity. The modified nanoparticles could disperse and self-assemble at the oil–water interface. Under the irradiation of a UV curing lamp, photoinitiator 819 decomposed to generate free radicals, which initiated the formation of free radical active chains from MMA monomers. Polymerization occurred, and polymerization was carried out on the HMX surface to form a PMMA shell. During the polymerization process, these C=C groups copolymerized with monomers to form a bonded polymer shell, and a PMMA-SiO_2_/TiO_2_ hybrid shell was formed to coat the HMX particles.

### 3.3. Crystalline Form Analysis

**Figure 6:**
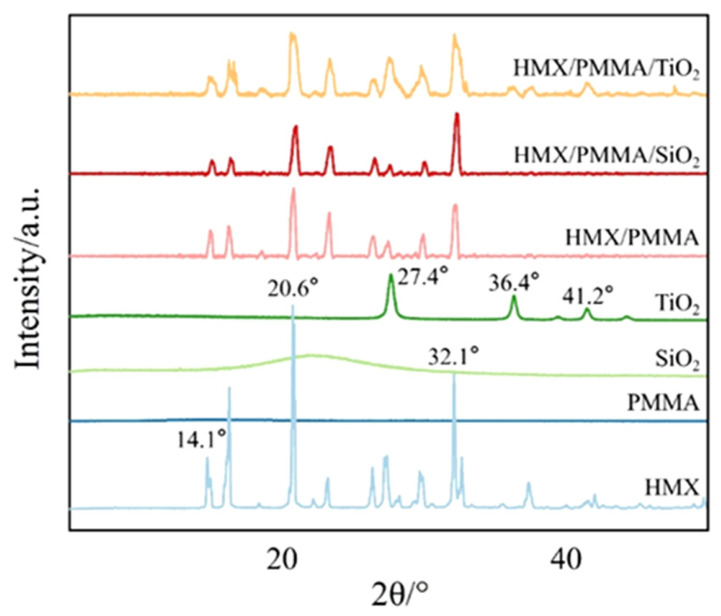
X-ray diffraction patterns of different samples.

Figure 6 shows the XRD patterns of raw HMX, PMMA, modifiers SiO_2_ and TiO_2_, HMX/PMMA, HMX/PMMA/SiO_2_, and HMX/PMMA/TiO_2_. Compared with raw HMX, the characteristic diffraction peaks of all samples at 15.1°, 20.6°, and 32.1° exhibit consistent shapes. Indicating that the crystalline structure of raw HMX did not change during the coating process, only the height of the diffraction peaks decreased and the width increased. This is because the binder PMMA and the modifiers SiO_2_ and TiO_2_ coated the explosive HMX particles to form microspheres, which enhanced the X-ray scattering phenomenon, leading to changes in the height and width of the diffraction peaks of the HMX-based composite energetic microspheres compared with raw HMX. After adding the modifiers SiO_2_ and TiO_2_, the XRD patterns of HMX/PMMA/modifier composite microspheres were a combination of those of HMX/PMMA and SiO_2_ or TiO_2_. The modifier SiO_2_ had an amorphous structure, and a broad diffraction band appeared obviously between 15° and 35°. However, due to the low addition amount of the modifier, the characteristic peaks in the XRD curve of HMX/PMMA/SiO_2_ were not obvious. The diffraction peaks of TiO_2_ at 27.5°, 36.5°, and 51.2° corresponded to the (1 1 0), (1 0 1), and (1 1 1) planes of the rutile crystal form, respectively, indicating that pure TiO_2_ had a rutile structure (PDF No. 21-1276) [17]. Meanwhile, the characteristic peaks of TiO_2_ also appeared in the HMX/PMMA/TiO_2_ sample, indicating that TiO_2_ was present in the composite microspheres.

### 3.4. Fourier Transform Infrared (FT-IR) Spectroscopy Analysis

**Figure 7:**
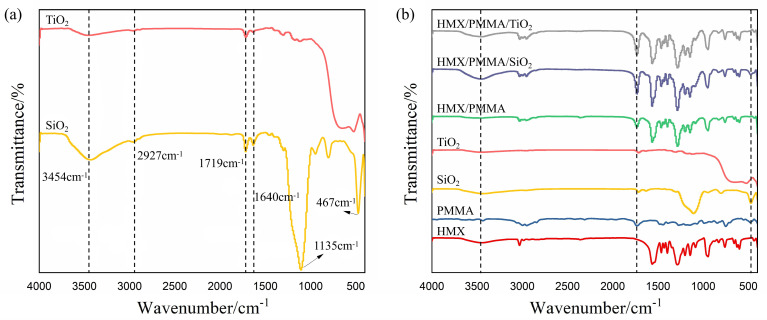
(**a**) Infrared spectrum of the modifier. (**b**) Infrared spectra of various materials.

The structure of the composite particles was further studied using FT-IR spectroscopy. It can be seen from Figure 7a that, under the modification of the coupling agent MPTMS, the C=O absorption peak of the coupling agent appeared at 1719 cm^−1^; the characteristic peak of C=C double bond stretching vibration of the coupling agent appeared at 1650 cm^−1^; and the absorption peaks of alkyl groups (CH_3_ or CH_2_) and surface hydroxyl groups of the coupling agent MPTMS also appeared at 2927 cm^−1^ and 3555 cm^−1^, indicating that the coupling agent MPTMS successfully modified SiO_2_ and TiO_2_.

In the FT-IR spectrum of HMX (Figure 7b), the main absorption peaks were in the range of 2900–3100 cm^−1^, showing a series of continuous absorption peaks. These absorption peaks were related to the -CH_2_ stretching vibration in HMX molecules and the O-H bond resonance in -NO_2_ groups. The N-NO_2_ characteristic peaks could be observed at 1569 cm^−1^ and 1258 cm^−1^, and the bending vibration characteristic peaks of -CH_2_- and -CH_3_ groups were shown in the range of 1550–1590 cm^−1^. The FT-IR spectrum of PMMA showed the vibration characteristic peak of -C-C- at 2850 cm^−1^ and the characteristic peak of C=O at 1730 cm^−1^. By comparing the FT-IR spectra of these three samples with that of raw HMX particles, it was found that the FT-IR spectra of the three HMX-based energetic microspheres all contained the stretching vibration characteristic peaks of explosive HMX particles and binder PMMA, indicating that the binder PMMA successfully coated the explosive HMX particles. The presence of these characteristic peaks provides clear evidence for the formation and composition of the composite microspheres. In addition, the modified microspheres showed absorption peaks of alkyl groups (CH_3_ or CH_2_) and surface hydroxyl groups at 2927 cm^−1^ and 3555 cm^−1^, indicating that the modified particles were present in the composite microspheres.

### 3.5. Thermal Performance Analysis

**Figure 8:**
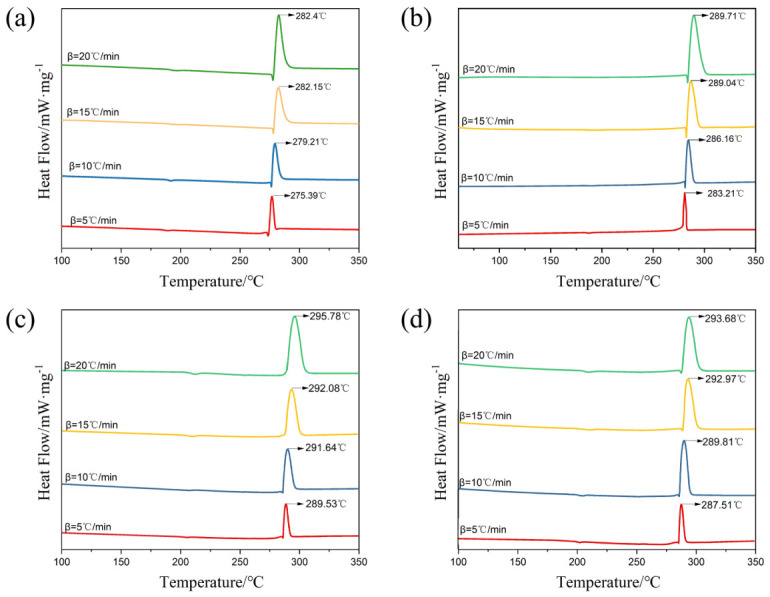
DSC curves of different samples. (**a**) Raw HMX, (**b**) HMA/PMMA, (**c**) HMX/PMMA/SiO_2_ (0.75%), (**d**) HMX/PMMA/TiO_2_ (0.5%).

The thermal performance of HMX-based composite microspheres was analyzed using a DSC-131 differential scanning calorimeter. The DSC curves of the obtained samples are shown in Figure 8.

As shown in Figure 8, raw HMX and HMX/PMMA composite energetic microspheres showed similar trends at four different heating rates. With a continuous increase in heating rate, the thermal decomposition peak temperature of each sample increased accordingly, showing a consistent trend. Further analysis revealed that the intensity of the endothermic peak in the thermal analysis curve of the energetic composite microspheres decreased after adding modified SiO_2_ and TiO_2_, while the endothermic peaks of raw HMX and HMX/PMMA microspheres without modifiers were very obvious, and the endothermic peaks of the composite microspheres disappeared. This is because the modifiers SiO_2_ and TiO_2_ in the composite microspheres are nanoscale materials with a large specific surface area. When heated, the temperature of the HMX/PMMA composite microspheres rises immediately without delay, resulting in an unobvious melting process.

By analyzing the exothermic peaks of raw HMX and HMX/PMMA composite microspheres during decomposition at four different heating rates, the Kissinger equations (Equations (2) and (3)) and the critical temperature of thermal explosion calculation equation (Equation (4)) were used to calculate the apparent activation energy (*Ea*), pre-exponential factor (*A*), peak temperature (*T_p_*_0_) of HMX when the heating rate approaches 0, and critical temperature of thermal explosion (*T_b_*) of each sample [18,19]. The calculated results are shown in Table 3.

Kissinger equations:

(2) $$\ln\left(\frac{\beta_{i}}{T_{p0}^{2}}\right)=\ln\left(\frac{AR}{Ea}\right)-\frac{Ea}{{RT}_{PI}}$$

(3) $$T_{pi}=T_{p0}+{a\beta }_{i}+{b\beta }_{i}^{3}+{c\beta }_{i}^{3}$$

Calculation equation for critical temperature of thermal explosion:

(4) $$T_{b}=\frac{Ea-\sqrt{{Ea}^{2}-4REaT_{p0}}}{2R}$$

where *β* is the heating rate (K/min or K/s); *T_p_*_0_ is the decomposition peak temperature of the explosive at heating rate *β*, in Kelvin (K); *A* is the pre-exponential factor, in min^−1^ or s^−1^; *R* is the gas constant, with a value of 8.315 J/(mol·K); *Ea* is the apparent activation energy, in J/mol.

**Figure 9:**
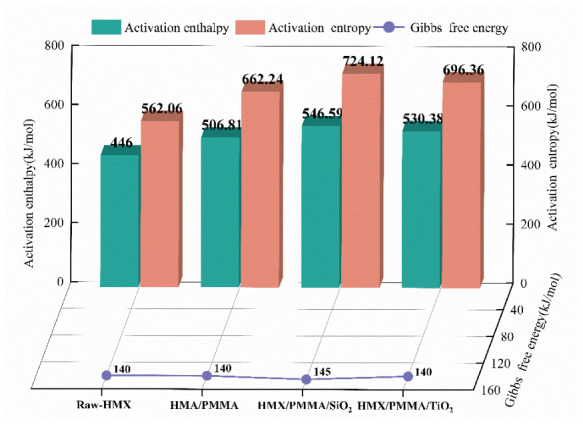
Thermodynamic parameters of each sample.

The data of Table 3 and Figure 9 show that the thermal decomposition kinetic parameters of HMX-based Polymer-Bonded Explosive (PBX) microspheres, such as apparent activation energy, pre-exponential factor, activation entropy, and activation enthalpy, increased to varying degrees, but the Gibbs free energy of the samples changed slightly. This can be attributed to the strong self-heating and autocatalytic effects of HMX, which are less affected by coating materials, resulting in a slight change in Gibbs free energy. Compared with HMX without binder, the thermal decomposition activation energies of HMX/PMMA energetic microspheres, HMX/PMMA/SiO_2_ composite energetic microspheres, and HMX/PMMA/TiO_2_ composite energetic microspheres increased by 52.32 kJ/mol, 79.86 kJ/mol, and 77.78 kJ/mol, respectively, significantly improving the thermal performance. This improvement is due to HMX being coated as the core material; PMMA decomposes at high temperature to generate a large number of free radicals, thereby improving the stability of free radicals and increasing the activation energy and thermal stability [20]. In contrast, the thermal stability of the composite microspheres with the modifiers SiO_2_ and TiO_2_ improved further. The modifiers SiO_2_ and TiO_2_ slowed down the decomposition rate of HMX during heating, thereby enhancing the thermal stability of the explosive HMX. Comprehensive analysis shows that the modifiers SiO_2_ and TiO_2_ both significantly improve the thermal stability of HMX/PMMA composite microspheres, and SiO2 exhibits a better modification effect.

### 3.6. Mechanical Sensitivity Analysis

According to GB/T 21567-2008 [21] and GB/T 21566-2008 [22] standard methods, the mechanical sensitivity and friction sensitivity of raw HMX, HMX/PMMA composite microspheres, HMX/PMMA/SiO_2_ composite microspheres, and HMX/PMMA/TiO_2_ composite microspheres were tested.

**Figure 10:**
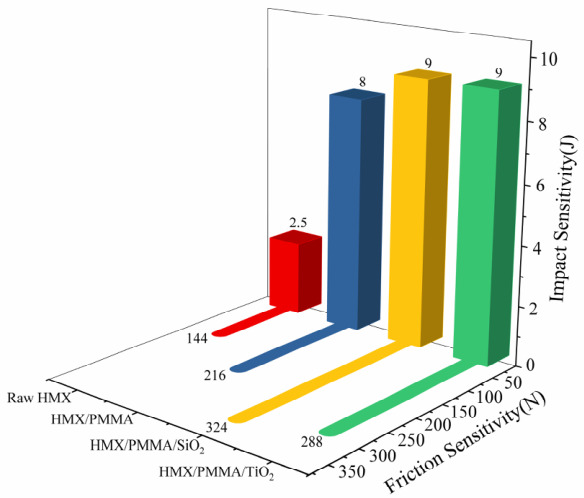
Mechanical sensitivity test results of different samples.

The test results are shown in Figure 10. The friction force and impact energy of raw HMX are 155 N and 2.5 J, respectively. The friction force and impact energy of the HMX/PMMA composite microspheres are 216 N and 8 J, respectively, and their mechanical sensitivity is significantly lower than that of raw HMX. The impact energies of HMX/PMMA/SiO_2_ and HMX/PMMA/TiO_2_ composite microspheres prepared via the photoinitiated emulsion method are both 9 J, which is 3.6 times higher than that of raw HMX. The friction forces are 325 N and 255 N, respectively, and the friction forces are 2.25 times and 2 times higher than that of raw HMX, indicating a significant improvement in safety. This indicates that the composite microspheres prepared via photoinitiated emulsion polymerization reduce the number of exposed crystal edges of HMX, making it difficult to induce hot spots [23]. The binder PMMA and modifiers (SiO_2_, TiO_2_) can play a buffering and protective role during the impact and friction of the composite microspheres, dispersing external impact energy, thereby significantly reducing the friction sensitivity and impact sensitivity of the composite microspheres.

Analysis of HMX/PMMA/modifier composite microspheres shows that the addition of nanoscale modifiers significantly improves the impact resistance and friction resistance of the microspheres. Owing to the small size and large specific surface area of nanoparticles, under external impact load, the force propagates along the surface of the nanoparticles and disperses to more surface areas [24], thereby reducing the force per unit area and the impact and friction sensitivity of the microspheres, which significantly improves the safety performance of the composite energetic materials.

### 3.7. Mechanical Property Analysis

**Figure 11:**
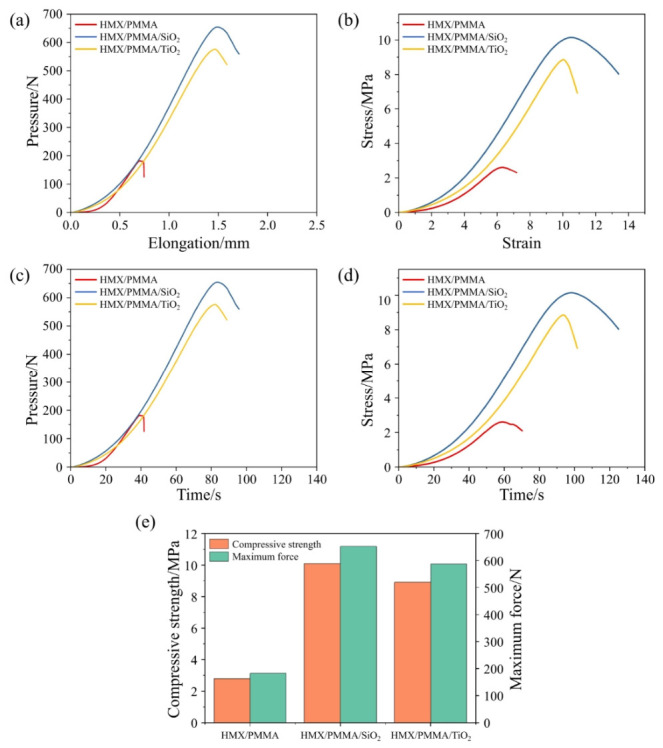
Static compression test curve of the sample drug column. (**a**) Pressure–elongation curve, (**b**) stress–strain curve, (**c**) pressure–time curve, (**d**) stress–time curve, (**e**) static mechanical properties.

A universal testing machine was used to test the static mechanical properties of HMX/PMMA, HMX/PMMA/SiO_2_, and RDX/PMMA/TiO_2_ explosive pellets. The compression performance parameters of the HMX-based PBX grains obtained from the tests are shown in Figure 11.

The data in Figure 11a–d show the variation patterns in the pressure–elongation curve, stress–strain curve, pressure–time curve, and stress–time curve of HMX-based PBXs in the static mechanical property test all show a trend of first increasing and then decreasing, and the test was stopped after the explosive pellets underwent quantitative deformation. Figure 11e shows the maximum pressure and compressive strength of the three coated microspheres, showing that the mechanical properties of the explosive pellets with SiO_2_ and TiO_2_ are improved to varying degrees compared with those of HMX/PMMA explosive pellets. The reason for this difference is that, during the static mechanical property test, interactions occur between particles. PMMA, as a binder, consumes part of the energy and plays a buffering role; however, its poor elasticity leads to weak mechanical properties of the composite samples. The addition of nano-SiO_2_ and TiO_2_ enhances the internal cross-linking degree between PMMA molecules, thereby improving the mechanical properties of the samples. Nano-SiO_2_ is uniformly dispersed in the PMMA macromolecular system and connected to the PMMA matrix through chemical bonds, which is conducive to stress transfer between nanoparticles and the matrix. When the material is stressed, it can disperse the energy that generates cracks and prevent the development of cracks, thereby improving the toughness and mechanical properties of the material [25,26,27]. The dispersibility of modified TiO_2_ and its cross-linking with the polymer PMMA matrix are improved. When subjected to external force, the shear stress around the particles is transferred, and the PMMA matrix undergoes local yielding, absorbing more energy to reach the mechanical equilibrium state of the composite material [27]. It can bear more loads, absorb more impact energy, and exhibit better reinforcement and toughening effects, thereby improving the toughness and strength of the matrix and further enhancing the mechanical properties of HMX-based PBXs.

## 4. Experimental Section

### 4.1. Materials

Raw HMX was purchased from Gansu Yinguang Chemical Industry Group Co., Ltd. (Baiyin, China). Methyl methacrylate (MMA) and photoinitiator 819 were purchased from Shanghai Macklin Biochemical Technology Co., Ltd. (Shanghai, China). (3-Mercaptopropyl) trimethoxysilane (MPTMS), pentaerythritol acrylate (PETRA), SiO_2_ (30 nm) and TiO_2_ (30 nm) were purchased from Shanghai Aladdin Biochemical Technology Co., Ltd. (Shanghai, China). Span-80, absolute ethanol, and poly(vinyl alcohol) (PVA) (the molecular weight of PVA is 47,000, and the degree of hydrolysis is 99%) were purchased from Tianjin Shengtai Chemical Reagent Co., Ltd. (Tianjin, China). Deionized water was prepared in the laboratory.

### 4.2. Preparation of Composite Particles

#### 4.2.1. Pretreatment Before Preparation

(1) Emulsification treatment of HMX surface

A certain amount of raw HMX and surfactant Span-80 (M_HMX_:M_Span-80_ = 99.9:0.1) were added to 50 mL of aqueous solution. This step could improve the dispersion of HMX in the aqueous solution, and emulsification was performed using an emulsifier at 6000 rpm for 10 min.

(2) Modification of SiO_2_ and TiO_2_

To obtain a stable water-in-oil (W/O) emulsion, (3-mercaptopropyl) trimethoxysilane (MPTMS) was used as a coupling agent to modify nano-SiO_2_ and TiO_2_ to change their surface hydrophilicity. Briefly, 0.1 mL of MPTMS was added to 30 mL of ethanol/water mixture under stirring and hydrolyzed at 20 °C for 6 h. Then, 10 g of nano-SiO_2_/TiO_2_ was added to 80 mL of ethanol/water mixture, and the hydrolyzed MPTMS solution was dropped into the nano-SiO_2_/TiO_2_ dispersion. The reaction was carried out at 55 °C for 25 h. The chemical reaction of modifying silica (SiO_2_) with MPTMS belongs to the silane coupling reaction. After the reaction, the mixture was centrifuged, and the collected precipitate was washed with water and ethanol. Finally, the obtained solid particles were vacuum-dried at 60 °C for 6 h to obtain modified nano-SiO_2_/TiO_2_ particles.

#### 4.2.2. Composite Microspheres Prepared by Photoinitiated Emulsion Polymerization

**Figure 12:**
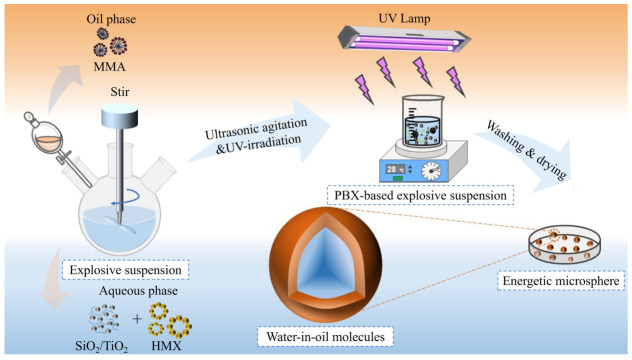
Technical route for synthesis.

The modified nano-SiO_2_/TiO_2_ was added to deionized water and subjected to ultrasonic treatment for 10 min. Then, the aqueous solution of HMX, PETRA, PVA, and photoinitiator 819 (each accounting for 1% of the mass of HMX) and composite emulsifier (M_Tween 80_: M_Span 80_ = 1:1) were added sequentially to prepare an aqueous phase solution. The oil phase solution (MMA, concentration 0.8%) was mixed with the aqueous phase solution, and emulsification was performed using an emulsifier at 15,000 rpm for 20 min to obtain a W/O emulsion. The emulsion was placed under a UV lamp (365 nm) and magnetically stirred for 10 min to initiate photocuring at room temperature. After polymerization, the HMX-based composite energetic microspheres were obtained by filtration, washing, and drying (Figure 12).

### 4.3. Characterization

The morphology and crystalline structure of the composite particles were analyzed using scanning electron microscopy (SEM), X-ray powder diffraction (XRD), and Fourier transform infrared spectroscopy (FT-IR). Thermal performance tests (DSC) of the composite particles were conducted using a differential scanning calorimeter at heating rates of 5, 10, 15, and 20 °C/min, with a sample mass of 0.5 ± 0.1 mg and a temperature range of 50–350 °C. Friction sensitivity and impact sensitivity were tested according to GB/T 21565-2008 [28] and GB/T 21567-2008 [21], respectively, at a test temperature of 20–25 °C and relative humidity of <80%. Finally, the samples were pressed into explosive pellets, and static mechanical properties were tested using a double-column electronic universal testing machine. The dimensions of the explosive pellets were Φ10 × 10 mm, with a test temperature of 20 °C, test speed of 0.5 mm/min, and humidity of 15%.

## 5. Conclusions

In this study, SiO_2_ and TiO_2_ were used as modifiers, and PMMA was used as a binder. An HMX/PMMA/modifier model was established via molecular dynamics to obtain the optimal theoretical ratio of composite particles. HMX/PMMA/modifier composite microspheres were successfully prepared via photoinitiated emulsion polymerization. The composite microspheres prepared under different process conditions were tested and characterized, and the modifier with the best effect and its content were identified. The addition of this modifier significantly improved the safety performance of the composite samples.

(1) Combining the results of molecular dynamics simulations and microsphere morphology analysis, the optimal addition amounts of the modifiers SiO_2_ and TiO_2_ in the HMX/PMMA/modifier composite system are 0.75% and 0.5%, respectively.
(2) Analysis of X-ray diffraction patterns and FT-IR spectra shows that the characteristic peaks of the prepared HMX/PMMA microspheres, HMX/PMMA/SiO_2_ composite microspheres, and HMX/PMMA/TiO_2_ composite microspheres are roughly the same as those of the XRD pattern and FT-IR spectrum of HMX. This indicates that the binder PMMA and modifiers successfully coated the explosive particles, and the crystalline form of HMX did not change.
(3) Thermal decomposition kinetics showed that the activation energy of HMX/PMMA/SiO_2_ and HMX/PMMA/TiO_2_ increased by 79.86 kJ/mol and 77.78 kJ/mol, respectively, significantly improving the thermal performance. The impact energies of HMX/PMMA/SiO_2_ and HMX/PMMA/TiO_2_ composite microspheres prepared via photoinitiated emulsion method are both 3.6 times higher than that of raw HMX. The friction forces are 2.25 times and 2 times higher than that of raw HMX, indicating a significant improvement in safety.
(4) The prepared composite microsphere samples were pressed into explosive pellets, and their static mechanical properties were evaluated. The results show that the addition of the modifiers SiO_2_ and TiO_2_ can significantly enhance the compressive capacity of HMX-based PBX pellets, with a significant improvement in mechanical properties. Meanwhile, the mechanical properties of HMX/PMMA composite microspheres also improved. Compared with HMX/PMMA microspheres without modifiers, the compressive strength increased by 7.3 MPa and 6.1 MPa, respectively.
(5) The addition of the modifiers SiO_2_ and TiO_2_ to HMX/PMMA microspheres improves the performance of the microspheres, and SiO_2_ exhibits the best modification effect. This solves the problem of poor mechanical properties of HMX/PMMA microspheres and provides a new idea for research on PMMA-coated energetic materials. Analysis of the above results shows that photoinitiated emulsion polymerization is an efficient desensitization technology for preparing composite microspheres with good morphology.

## Figures and Tables

**Table 1:** Mechanical properties of HMX/PMMA/SiO_2_ system.

Elastic Constant	HMX/ PMMA	HMX/ PMMA/ SiO_2_ (0.25%)	HMX/ PMMA/ SiO_2_ (0.5%)	HMX/ PMMA/ SiO_2_ (0.75%)	HMX/ PMMA/ SiO_2_ (1%)
Tensile modulus E/GPa	1.13	8.22	5.51	5.51	5.33
Bulk modulus K/GPa	1.93	2.23	5.81	6.00	6.18
Shear modulus G/GPa	1.50	1.60	1.33	1.80	2.33
Poisson’s ratio ν	0.67	0.35	0.55	0.57	0.69
Cauchy pressure C_12_-C_55_/GPa	1.02	1.28	3.56	5.35	0.59
K/G	1.37	1.50	5.36	3.32	2.65

**Table 2:** Mechanical properties of HMX/PMMA/TiO_2_ system.

Elastic Constant	HMX/ PMMA	HMX/ PMMA/ TiO_2_ (0.25%)	HMX/ PMMA/ TiO_2_ (0.5%)	HMX/ PMMA/ TiO_2_ (0.75%)	HMX/ PMMA/ TiO_2_ (1%)
Tensile modulus E/GPa	1.13	5.55	3.55	3.98	3.99
Bulk modulus K/GPa	1.93	6.05	5.59	5.16	6.09
Shear modulus G/GPa	1.50	1.36	1.03	1.28	1.26
Poisson’s ratio ν	0.67	0.52	0.65	0.55	0.55
Cauchy pressure C_12_-C_55_/GPa	1.02	3.59	3.59	2.99	3.91
K/G	1.37	5.55	5.31	5.03	5.85

**Table 3:** Thermal decomposition kinetic parameters of different samples.

Sample	Ea/(kJ·mol^−1^)	Log(A)	T_p0_/°C	T_b_/°C
Raw HMX	450.30	52.83	272.50	273.88
HMX/PMMA	502.62	58.09	282.31	283.75
HMX/PMMA/SiO_2_	530.16	59.26	290.55	291.28
HMX/PMMA/TiO_2_	528.08	59.25	287.96	289.28

## Data Availability

The original contributions presented in this study are included in the article. Further inquiries can be directed to the corresponding author(s).
